# Bacterial profile and antimicrobial susceptibility pattern of community and hospital-acquired urinary tract infections among UTI suspected geriatrics in Gondar town, Northwest Ethiopia

**DOI:** 10.1371/journal.pone.0323570

**Published:** 2025-05-16

**Authors:** Getachew Bitew Alebachew, Mulat Dagnew, Aklilu Ambachew, Belay Tessema

**Affiliations:** 1 Department of Medical Microbiology, School of Biomedical and Laboratory Sciences, College of Medicine and Health Sciences, University of Gondar, Gondar, Ethiopia; 2 Institute of Clinical Immunology, Faculty of Medicine, University of Leipzig, Leipzig, Germany; Tokyo Women's Medical University, JAPAN

## Abstract

**Background:**

Bacterial urinary tract infection (UTI) is the second most frequent infection next to respiratory tract infection within the geriatric population both in the community and hospital settings. There is a limited data regarding geriatrics UTI in this study area. Therefore, the current study aimed to assess the status of the community and hospital-acquired urinary tract infections, and antimicrobial susceptibility patterns among UTI suspected geriatrics which is essential to physicians and health care workers to implement appropriate intervention.

**Methods:**

A comparative cross-sectional study was conducted among 460 UTI suspected geriatrics admitted at the University of Gondar Comprehensive Specialized Hospital and attended in Gondar town (Kallen Bnakafl and Menna Geriatrics Support Center Clinics) from 1^st^ May 2022–14^th^ July 2022. Socio-demographic data were collected using structured questionnaires. Urine culture was performed and isolates were counted for significant growth by a colony counter, and their antibiotic susceptibility was done by the Kirby–Bauer disc diffusion method. Data were entered using Epi Data version 4.0.0 and analyzed by Stata/IC version 14.0. *P-*value < 0.05 at 95% CI was considered statistically significant.

**Result:**

The overall prevalence of UTI in geriatrics was 44.4%. The prevalence of UTI among community and hospitalized suspected patients was 38.7% and 50%, respectively. *Escherichia coli* (*E. coli)* (38.6%) predominated across the two groups, followed by *Klebsiella* spp. (15.8%), *S. saprophyticus* (12.2%), *P. mirabilis* (9.1%), *S. aureus* (5.9%), and *Citrobacter* spp. (2.8%). *Pseudomonas* spp. (7.1%), *K. rhinoscleromatis* (5.1%), and *P. vulgaris* (2.8%), were isolated from only hospitalized patients. Piperacillin-tazobactam susceptibly was 100% in both study groups. Nalidixic acid resistance was 50% to 87.5% and 50% to 100% in the isolates from community and hospitalized UTI suspects, respectively.

**Conclusion:**

This study found a high prevalence of bacterial UTI in geriatrics and a high rate of bacterial resistance was observed to most antimicrobial drugs tested. Piperacillin-tazobactam and meropenem were the most active antimicrobials for UTI. Therefore, expanding routine bacterial culture and antimicrobial susceptibility testing and strengthening regular surveillance systems are essential for appropriate patient care.

## Introduction

Urinary tract infections (UTIs) represent a significant global public health issue, second only to respiratory infections, and are a leading cause of morbidity and mortality worldwide [1,2]. Bacterial pathogens are the primary etiologic agents of UTIs, which can affect both the upper and lower urinary tracts, manifesting in symptoms like fever, dysuria, urgency, flank pain, burning sensations, suprapubic tenderness, and intermittent urination [3,4]. Urinary tract infections can be acquired in the community or hospital settings [5,6].

Community-acquired urinary tract infections (CA-UTIs) are defined as an infection contracted outside of a health care facility or present at the time of admission, primarily affecting individuals with uncomplicated UTIs caused by intestinal microbial flora [7,8]. In contrast, hospital-acquired urinary tract infections (HAUTIs) are an infection acquired in the hospital environment after 48–72 hours of hospital admission, often linked to indwelling urinary catheters, especially among the elderly or post-surgical patients, and those in intensive care units [9–11]. Although most UTIs are caused by a single pathogen, polymicrobial infections are more common in geriatrics, with two to five bacterial strains often involved. These infections typically arise when the host GI microbiota colonize the urethra ascends to the bladder then migrates to the kidney or prostate and an imbalance between the host responses and bacterial virulence [12–14].

Bacterial pathogens are responsible for over 95% of UTIs, with Gram-negative bacteria accounting for 80–85% of cases, and Gram-positive bacteria contributing to 15–20%. *Escherichia coli* is the most prevalent causative agent in both community and hospital settings [15,16]. In CA-UTIs, *E. coli* and *Klebsiella* spp. dominate, particularly among sexually inert geriatrics, causing 80% and 5–15% of cases respectively, while other Gram-negative and Gram-positive bacteria account for the remainder [3,17]. In HAUTIs, a wider range of pathogens is involved, including more resistant strains such as *E. coli, K. pneumoniae, P. aeruginosa*, *Staphylococci, Enterococci, Enterobacter, Proteus*, and others, often related to catheter use [18,19].

Antibiotic resistance poses a significant public health threat worldwide, including in Ethiopia [20], where there is a lack of research on the prevalence of UTI and antimicrobial resistance patterns in the geriatric’s population. While some studies have been conducted in Ethiopia, including in Gondar, on UTI prevalence and antimicrobial resistance in children and adults, data on the geriatrics is limited. Therefore, this study aimed to assess the prevalence of UTI and antimicrobial susceptibility patterns of bacteria isolated from hospitalized geriatric patients suspected of having UTI, compared to those in the community.

## Materials and methods

### Study design, period, and area

A comparative cross-sectional study was conducted from May 1 to July 14, 2022, at the University of Gondar Comprehensive Specialized Hospital (UoGCSH) and two geriatrics support centers in Gondar town: Kallen Bnakafl and Menna Geriatrics Support Centers (KBMGSC). Gondar town is located 742 km from Ethiopia’s capital, Addis Ababa, and 182 km from Bahir Dar, the Amhara Regional State capital. The UoGCSH serves approximately 13 million people and offers various medical services, including emergency care, anti-retroviral therapy (ART), chronic care, surgery, dental, pediatric, gynecologic, and obstetric services. Kallen Bnakafl provides permanent care for 210 individuals (pediatric, geriatric, and mentally challenged) and supports 474 individuals externally. Menna offers home-based care for 400 individuals and permanent care for 190.

### Study population

All geriatrics who attended at the UoGCSH and lived in Gondar town (KBMGSC) were used as the source population while all UTI suspected geriatrics admitted at the UoGCSH and attended in Gondar town (KBMGSC) were used as the study population. We have included geriatrics who admitted at the UoGCSH, and attended in Gondar town (KBMGSC clinics), having symptoms for UTI (such as; fever, vomiting, dysuria, back pain/tenderness, frequency or urgency), and who are informed. However, mentally challenging individuals and geriatrics who have been taking antibiotics in the last two weeks before and during the time of data collection period were excluded.

### Operational definitions

**Geriatric**: is aged group equal and above 65 years [17]. **UTI suspected geriatrics:** those geriatrics supposed by the physicians for having UTI and directed to the laboratory for microbiological analysis of urine in the hospital adult wards (C and D) and geriatric support center clinics.

### Sample size determination and sampling technique

The sample size was determined by using a double population proportion formula by considering the following statistical assumptions: 95% CI, taking the prevalence of UTI, P_1 _= 19.3% in the community and P_2 _= 30.7% in the hospital taken from the previous study in Dessie [21].


$$n=\frac{{\left(z_{\alpha ╱2}+z\beta \right)}^{2}\left(P_{1}\left(1-P_{1}\right)+P_{2}\left(1-P_{2}\right)\right)}{{\left(P_{1}-P_{2}\right)}^{2}}$$

Where: P_1_ was the prevalence of UTI in the community, and P_2_ was the prevalence of UTI in the hospital, Zα/2 = the critical value of the normal distribution curve at α/2 (for a confidence interval of 95%, α is 0.05 and the critical value is 1.96), Zβ = power (80%) = 0.84.


$$\text{n = }{\left(\text{1.96 + 0.84}\right)}^{\text{2}}\text{x }\left(\text{0.193}\left(\text{1- 0.193}\right)\text{ + 0.307}\left(\text{1- 0.307}\right)\right)\text{/ }{\left(\text{0.193- 0.307}\right)}^{\text{2}}\text{= 222.3 }\;\;\mathrm{\backslash textasciitilde}\;\;\text{ 223}$$

By considering a 5% non-response rate: 223 x 0.05 = 11.15 ~ 12, n_1 _= 223 + 12 = 235. Therefore, the final calculated sample size was equal to 235 for each and the total sample size was 470.

Simple random sampling technique (lottery) method was used to randomly include those geriatrics, whose age was ≥ 65 years, who had symptoms for UTI and attended at KBMGSC clinics. A complete list of geriatric patients obtained from the geriatrics support centers clinics medical record was used as a sampling frame.

The hospital study participants were drawn from the admitted geriatric patients who were on the adult wards (C and D) during the data collection period using a systematic random sampling method. The admission registration book was used to determine the average number of geriatrics patients admitted to the UoGCSH in each month of the previous year. Then simple random sampling (lottery) method was used to select the first number, and thereafter, patients were picked at a regular interval (sample interval) to meet the required sample size. The sample interval (K) was determined by dividing the average number of monthly patient’s flow (N) to each adult ward of the UoGCSH by the proportional sample size (n) given to the hospital (K = N/n).

Geriatrics in ward C were selected every K^th^ interval (3) after the first number (2) picked randomly from 1 to K. K = 600/230 = 2.61 ~ 3. Geriatrics in ward D were selected every K^th^ interval (2) after the first number (2) picked randomly from 1 to K. K = 450/230 = 1.96 ~ 2.

### Data collection and laboratory methods

The important socio-demographic and clinical variables of the study participants were collected by trained nurses via face-to-face interviews using a structured questionnaire (supplementary material). A sample of 15 ml freshly voided clean catch midstream urine was collected from each geriatric after they discard the initial flow of urine, in a sterile screw-capped, wide-mouth container labeled with unique sample number, date and time of collection. After sample collection, it was promptly transported and processed within 1–2 hours. If delayed, it was refrigerated at 2–8 °C for up to 6 hours in the Medical Bacteriology Laboratory at the University of Gondar College of Medicine and Health Sciences.

### Culture and bacterial identification

A calibrated wire loop (0.001 ml) was used to inoculate urine samples onto Cystine Lactose Electrolyte Deficient Medium (CLED) (BIOMARK^R^ Laboratories, India). After incubating at 37 °C for 18–24 hours, colonies were counted with a colony counter and multiplied by 1000 to assess significant growth. Bacterial growth of 10^5^ CFU/ml was considered significant for bacteriuria. Significant colonies were sub-cultured onto MacConkey and Mannitol Salt Agar (MSA) for species isolation. Identification of bacteria was done using colony characteristic, Gram reaction of the bacteria and biochemical tests following standard procedure. The Gram-negative bacterial species were identified by using different biochemical tests. The Gram-positive bacterial species were identified using colony characterization on MSA and novobiocin antibiotic test.

### Antimicrobial susceptibility testing

The antimicrobial susceptibility test for all identified bacterial isolates was conducted in vitro following the Clinical and Laboratory Standards Institute (CLSI) guidelines, using the Kirby-Bauer disk diffusion method on Muller-Hinton agar (MHA). A loop of 3–5 identical bacterial colonies was transferred to 5 ml of normal saline, mixed to form a homogeneous suspension, and adjusted to McFarland 0.5 turbidity for standardized inoculum size. The suspension was then swabbed onto MHA using a sterile cotton swab. Antimicrobial agents were selected based on their availability for UTI treatment and the 2022 CLSI guidelines [22]. The following antimicrobials were used: ampicillin (10 µg), amoxicillin-clavulanate (20/10 µg), tobramycin (10 µg), tetracycline (30 µg), ciprofloxacin (5 µg), ceftazidime (30 µg), cefazolin (30 µg), cefotaxime (30 µg), piperacillin-tazobactam (100/10 µg), meropenem (10 µg), nitrofurantoin (300 µg) and nalidixic acid (30 µg) for Enterobacteriaceae. For *Pseudomonas* species the antimicrobial disks were piperacillin-tazobactam (100/10 µg), gentamycin (10 µg), tobramycin (10 µg), norfloxacin (10 µg), meropenem (10 µg). Whereas, vancomycin (30 µg), norfloxacin (10 µg), penicillin (10 units), trimethoprim-sulfamethoxazole (1.25/23.75 µg), gentamycin (10 µg), doxycycline (30 µg) and novobiocin (5 µg) were for pathogenic Gram-positive cocci. The plates were incubated at 37 °C for 18–24 hours, and the inhibition zones around the discs were measured to the nearest millimeter using a ruler. The isolates were classified as susceptible, intermediate and resistant.

### Quality control

Culture media were prepared according to the manufacturer’s instructions, with sterility checked by incubating 5% of each batch at 35–37 °C overnight for contamination. Reference strains of *S. aureus* (ATCC-25923), *E. coli* (ATCC-25922), and *P. aeruginosa* (ATCC-27853) were used to check the performance of the media and antimicrobials.

### Data processing and analysis

Data were coded and entered into Epi Data version 4.6.0.0, with completeness and validity checks. It was then exported to Stata/IC version 14.0 for analysis. Summary statistics described the study participants, with data presented in text, tables, and figures. A p-value of < 0.05 was considered statistically significant.

### Ethical approval

Ethical clearance was obtained from Research and Ethical Review Committee of School of Biomedical and Laboratory Sciences, College of Medicine and Health Sciences, University of Gondar (Ref. No 204). A letter of permission was also obtained from the UoGCSH Clinical Director, KBMGSC Administration Offices. The study participants were informed about the process, its purpose, and their right to refuse the study. A written informed consent was signed by study subjects. Codes were used to represent the study participants so that only authorized personnel could access the data. Geriatrics who had positive bacteriological results were linked to health professionals for the required care.

## Results

### Socio-demographic and clinical characteristics of the study participants

In this study, 460 out of the expected 470 participants were eligible and included, yielding a response rate of 97.9%. Of which, the highest number 377/460 (82.0%) of study participants were female and their age was ranged from 65–100 years with mean age of 71.0 ± 7.25 SD years. The majority of the participants 248/460 (53.9%) were in the age group of 65–75 and 325/460 (70.7%) (*P*-value = **0.014**) of them had not get the chance of formal education. Due to their age and health problem, most of this study participants 283/460 (61.5%) (*P*-value = **0.037**) had no a job and half of the study participants 230/460 (50%) had no any monthly income. According to their residence, the majority 316/460 (68.7%) of the participants live in urban settings as shown in (Table 1).

**Table 1:** Socio-demographic and clinical characteristics of the study participants in Gondar town, May 1 to July 14, 2022.

Characteristics (n = 460)		Community, 230 (50%)	Hospital, 230 (50%)	Total, 460 (100%)	*P*-value
Gender	Male	42 (18.3)	41 (17.8)	83 (18.0)	–
	Female	188 (81.7)	189 (82.2)	377 (82.0)	**0.034**
Age (years)	65–75	134 (58.3)	114 (49.6)	248 (53.9)	–
	76–85	66 (28.7)	86 (37.4)	152 (33.0)	0.457
	≥ 86	30 (13.0)	30 (13.0)	60 (13.0)	0.911
Education Level	No formal education	164 (71.3)	161 (70.0)	325 (70.7)	**0.014**
	Primary level	37 (16.1)	44 (19.1)	81 (17.6)	0.313
	Secondary level and above	29 (12.6)	25 (10.9)	54 (11.7)	–
Residence	Urban	230 (100)	86 (37.4)	316 (68.7)	0.863
	Rural	0 (0)	144 (62.6)	144 (31.3)	0.692
Income status (ETB)	No monthly income	230 (100)	0 (0)	230 (50.0)	0.631
	1000–2000	0 (0)	75 (32.6)	75 (16.3)	0.236
	2001–3000	0 (0)	61 (26.5)	61 (13.3)	0.116
	3001–4000	0 (0)	49 (21.3)	49 (10.7)	0.152
	≥ 4001	0 (0)	45 (19.6)	45 (9.8)	–
Occupation	Private employee	0 (0)	22 (9.6)	22 (4.8)	0.692
	House wife	0 (0)	63 (27.4)	63 (13.7)	**0.005**
	Merchant	0 (0)	28 (12.2)	28 (6.1)	0.556
	No job	194 (84.4)	89 (38.7)	283 (61.5)	**0.037**
	Others	36 (15.7)	28 (12.2)	64 (13.9)	–
Previous UTI	Yes	134 (58.3)	162 (70.4)	296 (64.4)	**0.040**
	No	96 (41.7)	68 (29.6)	164 (35.7)	–
Recurrent UTI	No recurrence	192 (83.5)	144 (62.6)	336 (73.0)	–
	≥ 2 per 6 months	21 (9.1)	40 (17.4)	61 (13.3)	0.608
	≥ 3 per 12 months	17 (7.4)	46 (20.0)	63 (13.7)	0.248
Previous catheterization	Yes	18 (7.8)	103 (44.8)	121 (26.3)	**0.001**
	No	212 (92.2)	127 (55.2)	339 (73.7)	–
Antibiotic use without prescription	Yes	104 (45.2)	149 (64.8)	253 (55.0)	0.360
	No	126 (54.8)	81 (35.2)	207 (45.0)	–
Diabetes Miletus	Yes	33 (14.4)	79 (34.4)	112 (24.4)	**0.014**
	No	197 (85.7)	151 (65.7)	348 (75.7)	–
Hospitalization (days)	2 days	0 (0)	42 (18.3)	42 (9.1)	–
	3 days	0 (0)	26 (11.3)	26 (5.7)	0.768
	4 days	0 (0)	39 (17.0)	39 (8.5)	0.106
	≥ 5 days	0 (0)	123 (53.5)	123 (26.7)	**0.044**

Key:- ETB: Ethiopian birr; others types of job: farmer, cultural drink (tela, tej and areki) workers and food handler.

Among the common risk factors for UTIs examined in this study, a history of previous UTIs and antibiotics use without prescriptions were reported by 296/460 (64.4%) and 253/460 (55.0%) participants, respectively. Notably, among those who reported using antibiotics without a prescription, the majority 149/253 (58.9%) were HAUTI developed patients. Additionally, majority of the participants did not have a history of catheter insertion 339/460 (73.7%) (*P*-value = **0.001**) and diabetes Miletus 348/460 (75.7%) (*P*-value = **0.014**) (Table 1).

### Prevalence of urinary tract infection

The overall prevalence of UTI in this study was 204/460 (44.4%) (95% CI: 39.79–48.90). Out of the total UTI detected, the magnitude of HAUTI was significantly higher (*P* = **0.015**), 115/230 (50%) (95% CI: 43.49–56.51) when compared with that of the CA-UTI, 89/230 (38.7%) (95% CI: 32.35–45.04) in this study (Table 2).

**Table 2:** Distribution of culture among CA-UTI versus HAUTI suspects in Gondar town, May 1 to July 14, 2022.

Types of UTIs	Urine cultured	Cultured positive cases	Percentage	X^2^	*P*
CA-UTI	230	89	38.70		
HAUTI	230	115	50.00	5.954	**0.015**
Total	460	204	44.4		

Key:- Chi-squire, X^2^ = 5.954, df = 1, *P* = 0.015; HAUTI: hospital-acquired UTI; CA-UTI: community-acquired UTI.

**Fig 1:**
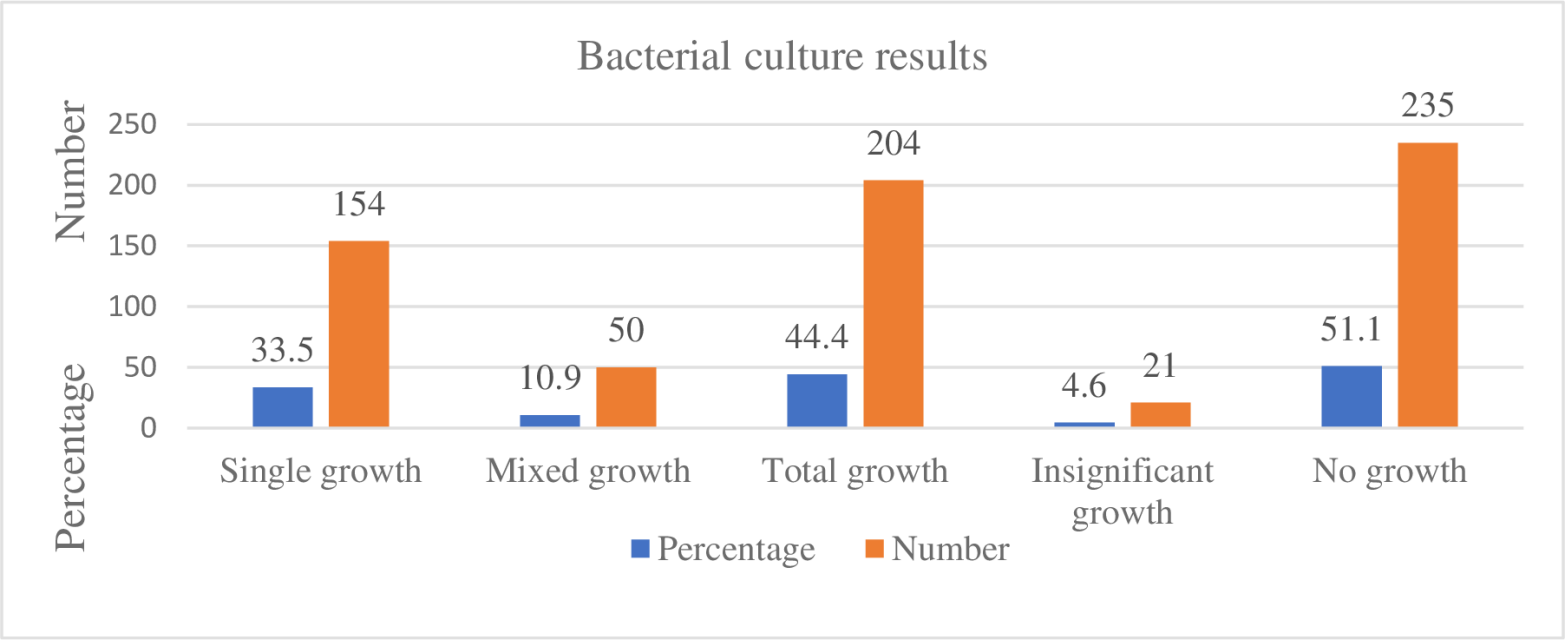
Bacterial culture results after urine samples processed in UTI suspected geriatrics from May 1 to July 14, 2022.

Of the total 460 urine specimens processed, 235/460 (51.1%) showed no bacteriuria growth and 21/460 (4.6%) showed insignificant bacterial growth. Significant growth was found in 204/460 (44.4%) of the samples with 154/460 (33.5%) being single growth and 50/460 (10.9%) being mixed growth with two organisms in hospitalized patients. Fifty of four hundred sixty (10.9%) samples with two bacteria each were isolated, making the number of bacteria isolated to be 254/460 with the isolation rate of 55.2%. From a total of 254 different isolated bacterial uropathogens, 165/254 (65.0%) were hospital-acquired setting isolates, and 89/254 (35.0%) were community-acquired setting isolates. There was 206/254 (81.1%) Gram-negative and 48/254 (18.9%) Gram-positive bacteria isolated (Fig 1).

**Fig 2:**
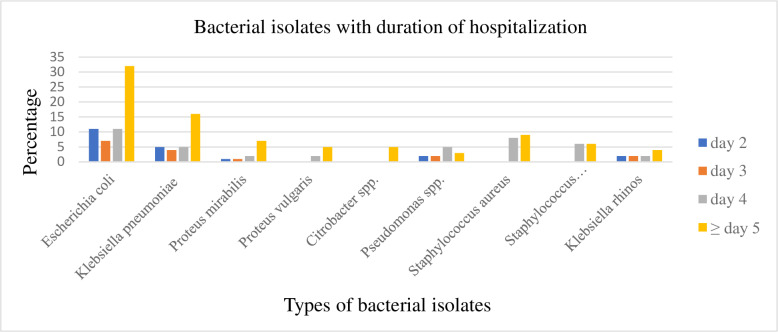
Distribution of bacterial isolates with the duration of hospitalization at Gondar Comprehensive Specialized Hospital from May 1 to July 14, 2022.

The frequency of isolated bacteria increases when days stayed in the hospital increases. *Escherichia coli*, *K. pneumoniae*, *K. rhinoscleromatis*, *P. mirabilis, P. vulgaris, Citrobacter* spp., and *S. aureus* were increased more after the fourth days of hospitalization. However, *S. saprophyticus* was found during the fourth and after the fourth days of hospitalization equally and *Pseudomonas* spp. was prevailed started from the fourth day of hospitalization (Fig 2).

The predominant bacteria isolated in both CA-UTI and HAUTI were *E. coli* 48/89 (53.9%) versus 61/165 (37.0%) (*P*-value = **0.023**), followed by *K. pneumoniae* 16/89 (18.0%) vs. 30/165 (18.2%), *S. saprophyticus* 13/89 (14.6%) vs. 12/165 (7.3%), *S. aureus* 6/89 (6.7%) vs. 17/165 (10.3%), *P. mirabilis* 4/89 (4.5%) vs. 11/165 (6.7%), and *Citrobacter* spp. 2/89 (2.3%) vs. 5/165 (3.0%) were isolated in both study groups whereas *Pseudomonas* spp. 12/165 (7.3%) (*P*-value = **0.0049**), *K. rhinoscleromatis* 10/165 (6.1%) (*P*-value = **0.015**) and *P. vulgaris* 7/165 (4.2%) were isolated only in hospitalized patients (Table 3).

**Table 3:** Distribution of bacterial isolates among CA-UTI and HAUTIs in Gondar town, May 1 to July 14, 2022.

Types of UTIs					
Isolated organisms	CA-UTI (n = 89)	HAUTI (n = 165)		Total	*P*-value
		Isolate 1	Isolate 2		
*Escherichia coli*	48 (53.9%)	49 (42.6%)	12 (24%)	109 (42.9%)	**0.023**
*K. pneumoniae*	16 (18%)	23 (20%)	7 (14%)	46 (18.1%)	1.000
*K. rhinoscleromatis*	0 (0%)	6 (5.2%)	4 (8%)	10 (3.9%)	**0.015**
*P. mirabilis*	4 (4.5%)	9 (7.8%)	2 (4%)	15 (5.9%)	0.582
*P. vulgaris*	0 (0%)	4 (3.5%)	3 (6%)	7 (2.8%)	0.051
*Pseudomonas* spp.	0 (0%)	7 (6.1%)	5 (10%)	12 (4.7%)	**0.0049**
*Citrobacter* spp.	2 (2.3%)	2 (1.7%)	3 (6%)	7 (2.8%)	1.000
*S. aureus*	6 (6.7%)	10 (8.7%)	7 (14%)	23 (9.1%)	1.000
*S. saprophyticus*	13 (14.6%)	5 (4.4%)	7 (14%)	25 (9.8%)	0.122
Total	89 (100%)	115 (100%)	50 (100%)	254 (100%)	

Key:- HAUTI: hospital-acquired UTI; CA-UTI: community-acquired UTI

### Antimicrobial susceptibility of community and hospital-acquired urinary tract infections

In this study, the antimicrobial susceptibility pattern of the bacterial isolates was indifferent between hospital and community-based geriatric participants. *Klebsiella pneumoniae* was resistant to 100% for ampicillin and 90% for tetracycline in HAUTI and 87.5% for nalidixic acid in CA-UTI. Nitrofurantoin, ampicillin, and tetracycline were resisted 100% by *P. mirabilis* in HAUTI and tetracycline was 100% resisted in CA-UTI. *Citrobacter* spp. was 100% resistant to nalidixic acid and ceftazidime in HAUTI. *Pseudomonas* spp. was 100% resistant to gentamycin and tobramycin in HAUTI. Nalidixic acid and tetracycline were resisted 96.7% vs 75% and 88.5% vs 75% by *E. coli* isolates in HAUTI and CA-UTI, respectively. *Staphylococcus aureus* isolates were resistant 82.4% vs. 66.7% to penicillin in HAUTI and CA-UTI, respectively (Tables 4 and 5).

**Table 4:** Antimicrobial susceptibility pattern of isolates from HAUTI suspected geriatrics in Gondar town, May 1 to July 14, 2022.

Isolates (N = 165)		Antibiotics													
Gram-negative		AMP n(%)	AMC n(%)	TOB n(%)	TET n(%)	GEN n(%)	NOR n(%)	CPR n(%)	CAZ n(%)	CZN n(%)	CTX n(%)	TZP n(%)	MRP n(%)	NIT n(%)	NAL n(%)
*Escherichia coli* n = 61	S	15(24.6)	15(24.6)	42(68.9)	7(11.5)	ND	ND	47(81)	27(44.3)	17(27.9)	30(49.2)	61(100)	49(80.3)	45(73.8)	2(2.38)
	I	7(11.5)	7(11.5)	6(9.84)	0(0)	ND	ND	5(11.9)	0(0)	2(2.4)	3(4.92)	0(0)	4(6.56)	3(4.92)	0(0)
	R	39(63.9)	39(63.9)	13(21.3)	54(88.5)	ND	ND	9(7.14)	34(55.7)	42(68.9)	28(45.9)	0(0)	8(13.1)	13(21.3)	59(96.7)
*K. pneumoniae* n = 30	S	0(0)	19(63.3)	17(56.7)	3(10)	ND	ND	25(83.3)	9(30)	13(43.3)	15(50)	30(100)	26(86.7)	25(83.3)	5(16.7)
	I	0(0)	1(3.33)	2(6.67)	0(0)	ND	ND	0(0)	0(0)	2(6.67)	2(6.67)	0(0)	2(6.67)	0(0)	0(0)
	R	30(100)	10(33.3)	11(36.7)	27(90)	ND	ND	5(16.7)	21(70)	15(50)	13(43.3)	0(0)	2(6.67)	5(16.7)	25(83.3)
*K. rhinoscler-omatis* n = 10	S	2(20)	3(30)	5(50)	2(20)	ND	ND	5(50)	9(90)	6(60)	6(60)	10(100)	10(100)	6(60)	5(50)
	I	0(0)	0(0)	1(10)	0(0)	ND	ND	2(20)	0(0)	1(10)	1(10)	0(0)	0(0)	0(0)	0(0)
	R	8(80)	7(70)	4(40)	8(80)	ND	ND	3(10)	1(10)	3(30)	3(30)	0(0)	0(0)	4(40)	5(50)
*P. mirabilis* n = 11	S	0(0)	4(36.4)	8(72.7)	0(0)	ND	ND	4(36.4)	8(72.7)	6(54.6)	5(45.5)	11(100)	11(100)	0(0)	2(18.2)
	I	0(0)	3(27.3)	1(9.09)	0(0)	ND	ND	1(9.09)	0(0)	0(0)	1(9.09)	0(0)	0(0)	0(0)	0(0)
	R	11(100)	4(36.4)	2(18.2)	11(100)	ND	ND	6(54.6)	3(27.3)	5(45.5)	5(45.5)	0(0)	0(0)	11(100)	9(81.8)
*P. vulgaris* n = 7	S	0(0)	2(28.6)	6(85.7)	2(28.6)	ND	ND	3(42.9)	2(28.6)	3(42.9)	4(57.1)	7(100)	4(57.1)	5(71.4)	0(0)
	I	1(14.3)	2(28.6)	0(0)	0(0)	ND	ND	1(14.3)	0(0)	1(14.3)	1(14.3)	0(0)	1(14.3)	0(0)	0(0)
	R	6(85.7)	3(42.9)	1(14.3)	5(71.4)	ND	ND	3(42.9)	5(71.4)	3(42.9)	2(28.6)	0(0)	2(28.6)	2(28.6)	7(100)
*Citrobacter* spp. n = 5	S	3(60)	3(60)	3(60)	2(40)	ND	ND	4(80)	0(0)	3(60)	1(20)	5(100)	5(100)	3(60)	0(0)
	I	2(40)	2(40)	2(40)	3(60)	ND	ND	1(20)	0(0)	2(40)	4(80)	0(0)	0(0)	2(40)	0(0)
	R	0(0)	0(0)	0(0)	0(0)	ND	ND	0(0)	5(100)	0(0)	0(0)	0(0)	0(0)	0(0)	5(100)
Total n = 124	S	20(16.1)	46(37.1)	81(65.3)	16(12.9)	–	–	88(71.0)	55(44.4)	48(38.7)	61(49.2)	124(100)	105(84.7)	84(67.7)	14(11.3)
	I	10(6.45)	15(11.3)	12(9.67)	3(2.42)	–	–	10(8.07)	0(0)	8(6.45)	12(9.67)	0(0)	7(5.65)	5(4.03)	0(0)
	R	94(77.4)	63(51.6)	31(25)	105(84.7)	–	–	26(21.0)	69(55.7)	68(54.8)	51(41.1)	0(0)	12(9.68)	35(28.2)	110(88.7)
*P*seudomonas spp. n = 12	S	ND	ND	0(0)	ND	0(0)	3(25)	ND	ND	ND	ND	12(100)	6(50)	ND	ND
	I	ND	ND	0(0)	ND	0(0)	0(0)	ND	ND	ND	ND	0(0)	0(0)	ND	ND
	R	ND	ND	12(100)	ND	12(100)	9(75)	ND	ND	ND	ND	0(0)	6(50)	ND	ND
**Gram-positive**		**VAN n(%)**	**PEN n(%)**	**SXT n(%)**	**DOX n(%)**	**GEN n(%)**	**NOR n(%)**	**NV** **n(%)**							
*S. aureus* n = 17	S	10(58.8)	3(25)	12(70.6)	10(58.8)	15(88.2)	12(70.6)	ND							
	I	2(11.8)	0(0)	1(5.88)	0(0)	2(11.8)	0(0)	ND							
	R	5(29.4)	14(82.4)	4(23.5)	7(41.2)	0(0)	5(29.4)	ND							
*S. saprophyticus* n = 12	S	7(58.3)	6(50)	8(66.7)	9(75)	9(75)	7(58.3)	0(0)							
	I	1(8.3)	0(0)	0(0)	3(25)	3(25)	1(8.3)	0(0)							
	R	4(33.3)	6(50)	4(33.3)	0(0)	0(0)	4(33.3)	14(100)							
Total n = 29	S	17(58.6)	9(31)	20(69)	19(65.5)	24(82.8)	19(65.5)	–							
	I	3(10.4)	0(0)	1(3.5)	0(0)	5(17.2)	1(3.5)	–							
	R	9(31)	20(69)	8(27.6)	10(34.5)	0(0)	9(31)	–							

Key:- AMP: ampicillin; AMC: amoxicillin-clavulanate; TOB: tobramycin; TET: tetracycline; CPR: ciprofloxacin; CAZ: ceftazidime; CZN: cefazolin; CTX: cefotaxime; TZP: piperacillin-tazobactam; MRP: meropenem; NIT: nitrofurantoin; NAL: nalidixic acid; GEN: gentamycin; NOR: norfloxacin; VAN: vancomycin; PEN: penicillin; SXT: trimethoprim-sulfamethoxazole; DOX: doxycycline; NV: novobiocin and ND: not-done.

**Table 5:** Antimicrobial susceptibility pattern of isolates from CA-UTI suspected geriatrics in Gondar town, May 1 to July 14, 2022.

Isolates (N = 89)		Antibiotics													
Gram-negative		AMP n(%)	AMC n(%)	TOB n(%)	TET n(%)	GEN n(%)	NOR n(%)	CPR n(%)	CAZ n(%)	CZN n(%)	CTX n(%)	TZP n(%)	MRP n(%)	NIT n(%)	NAL n(%)
*Escherichia coli* n = 48	S	22(45.8)	40(83.3)	39(81.3)	8(16.7)	ND	ND	38(79.2)	26(54.2)	23(47.9)	36(75)	48(100)	46(95.8)	34(70.8)	10(20.8)
	I	3(6.25)	3(6.25)	2(4.17)	4(8.33)	ND	ND	2(4.17)	2(4.17)	2(4.17)	0(0)	0(0)	2(4.17)	2(4.17)	2(4.17)
	R	23(47.9)	5(10.4)	7(14.6)	36(75)	ND	ND	8(16.7)	20(41.7)	23(47.9)	12(25)	0(0)	0(0)	12(25)	36(75)
*K. pneumoniae* n = 16	S	5(31.3)	14(87.5)	11(68.8)	10(62.5)	ND	ND	13(81.3)	5(31.3)	7(43.8)	8(50)	16(100)	16(100)	11(68.8)	2(12.5)
	I	1(6.25)	1(6.25)	2(12.5)	1(6.25)	ND	ND	1(6.25)	2(12.5)	0(0)	0(0)	0(0)	0(0)	2(12.5)	0(0)
	R	10(62.5)	1(6.25)	3(18.8)	5(31.3)	ND	ND	2(12.5)	9(56.3)	9(56.3)	8(50)	0(0)	0(0)	3(18.8)	14(87.5)
*P. mirabilis* n = 4	S	2(50)	3(75)	3(75)	0(0)	ND	ND	2(50)	1(25)	1(25)	1(25)	4(100)	4(100)	3(75)	1(25)
	I	0(0)	0(0)	0(0)	0(0)	ND	ND	1(25)	1(25)	1(25)	0(0)	0(0)	0(0)	0(0)	0(0)
	R	2(50)	1(25)	1(25)	4(100)	ND	ND	1(25)	2(50)	2(50)	3(75)	0(0)	0(0)	1(25)	3(75)
*Citrobacter* spp. n = 2	S	1(50)	1(50)	1(50)	1(50)	ND	ND	1(50)	0(0)	1(50)	1(50)	2(100)	2(100)	1(50)	1(50)
	I	1(50)	1(50)	1(50)	1(50)	ND	ND	1(50)	0(0)	1(50)	1(50)	0(0)	0(0)	1(50)	0(0)
	R	0(0)	0(0)	0(0)	0(0)	ND	ND	0(0)	2(100)	0(0)	0(0)	0(0)	0(0)	0(0)	1(50)
Total n = 70	S	30(42.9)	58(82.9)	54(77.1)	19(27.1)	–	–	54(77.1)	31(44.3)	32(45.7)	46(65.7)	70(100)	68(97.1)	49(70)	13(18.6)
	I	5(7.14)	5(7.14)	5(7.14)	6(8.57)	–	–	5(7.14)	6(8.57)	4(5.71)	1(1.43)	0(0)	2(2.86)	5(7.14)	3(4.29)
	R	35(50)	7(10)	11(15.7)	45(64.3)	–	–	11(15.7)	33(47.1)	34(48.6)	23(32.9)	0(0)	0(0)	16(22.9)	54(77.1)
**Gram-positive**		**VAN n(%)**	**PEN n(%)**	**SXT n(%)**	**DOX n(%)**	**GEN n(%)**	**NOR n(%)**	**NV** **n(%)**							
*S. aureus* n = 6	S	4(66.7)	1(16.7)	3(50)	6(100)	6(100)	4(66.7)	ND							
	I	0(0)	1(16.7)	0(0)	0(0)	0(0)	1(16.7)	ND							
	R	2(33.3)	4(66.7)	3(50)	0(0)	0(0)	1(16.7)	ND							
*S. saprophyticus* n = 13	S	9(69.2)	7(53.9)	10(76.9)	12(92.3)	13(100)	9(69.2)	0(0)							
	I	1(7.69)	1(7.69)	3(23.1)	1(7.69)	0(0)	1(7.69)	0(0)							
	R	3(23.1)	5(38.5)	0(0)	0(0)	0(0)	3(23.1)	13(100)							
Total n = 19	S	13(68.4)	8(42.1)	13(68.4)	18(94.7)	19(100)	13(68.4)	–							
	I	1(5.26)	2(10.5)	4(21.1)	1(5.26)	0(0)	2(10.5)	–							
	R	5(26.3)	9(47.4)	2(10.5)	0(0)	0(0)	4(21.1)	–							

Key:- AMP: ampicillin; AMC: amoxicillin-clavulanate; TOB: tobramycin; TET: tetracycline; CPR: ciprofloxacin; CAZ: ceftazidime; CZN: cefazolin; CTX: cefotaxime; TZP: piperacillin-tazobactam; MRP: meropenem; NIT: nitrofurantoin; NAL: nalidixic acid; GEN: gentamycin; NOR: norfloxacin; VAN: vancomycin; PEN: penicillin; SXT: trimethoprim-sulfamethoxazole; DOX: doxycycline; NV: novobiocin and ND: not-done.

*Escherichia coli*, *K. pneumoniae, P. mirabilis,* and *Citrobacter* spp. were susceptible to 100% for piperacillin-tazobactam in both CA-UTI and HAUTI. *Proteus vulgaris*, *K. rhinoscleromatis,* and *Pseudomonas* spp. were susceptible to 100% for piperacillin-tazobactam in HAUTI. *Proteus mirabilis, K. rhinoscleromatis* and *Citrobacter* spp. in HAUTI, and *P. mirabilis, K. pneumoniae* and *Citrobacter* spp. in CA-UTI (100%) were susceptible to meropenem. For both types of UTI piperacillin-tazobactam (100%) was the most sensitive drug (Tables 4 and 5).

## Discussion

Urinary tract infection is the second most common infectious disease in the geriatric population in both community and hospital settings. The infection often leads to the development of urinary stones and finally may cause kidney failure if it remains untreated [23]. It is seen more commonly in geriatrics than young individuals related to an altered immune function, frequent and longer catheterization, co-morbidity, malnutrition, poor emptying of the bladder, loss of estrogen in postmenopausal women, prostatic hypertrophy and loss of the bactericidal activity of prostatic secretions in men, and an increased likelihood of hospitalization [3,24].

In the current study, the overall prevalence of UTI was 44.4%. This finding lies between the high prevalence of 90.1% in Ethiopia-Shashemene [25], and the low prevalence of 0.7% in USA [26], in different parts of the world. The results of this study also showed that the etiologic agents of UTIs mainly belonged to Gram-negative bacilli (81.1%) more than Gram-positive cocci (18.9%). The possible reasons might be due to differences in laboratory methods and uropathogens causing a UTI originate from the host gastro-intestine, which contains many Gram-negative organisms. Bacterial etiologies of UTIs can also vary across regions and over time within a population.

In the present study, the prevalence of HAUTI and CA-UTI were 50.0% and 38.7%, respectively. This prevalence was comparable with Ethiopia-Addis Ababa, (51.4%) HAUTI [27], Pakistan, (44%) CA-UTI [28] and Ethiopia-Harar (52.6% vs. 30.3%) [29]. In contrast, the result of this study was higher than studies done in USA (4% vs. 0.7%) [26], Saudi Arabia, HAUTI (6.5%) [30], India, CA-UTI (19.6%) [31], Nigeria (14.9% vs. 11.1%) [8], Italy, HAUTI (31%) [6], Uganda, CA-UTI (32.2%) [32], Ethiopia-Bahir Dar, HAUTI (9.4%) [33], Ethiopia-Metu, CA-UTI (16.7%) [34], and Ethiopia-Dessie (30.7% vs. 19.3%) [21]. On the other hand, this findings showed a lower prevalence of UTI than reports in Democratic Republic of Congo, HAUTI (65%) [35], Bangladesh, CA-UTI (45.5%) [16], and Chad, HAUTI (70.4%) [36], Egypt, CA-UTI (66%) [37], respectively. The reality might justify the variation that differences exist in the study setting, study period, sample size, socio-demographic variations and laboratory methods.

In the present study, hospitalized geriatrics had a higher prevalence of UTI (50.0%) than the community (38.70%). It was similar to studies that were conducted in Bangladesh (50% vs. 45%) [18] and Ethiopia-Harar (52.6% vs. 30.3%) [29] HAUTI and CA-UTI, respectively. This could be due to hospitalized geriatrics exposed to inappropriate catheter insertion technique and longer duration of catheterization which facilitates the entry of the pathogen to the urinary tract. Other possible reasons might be due to longer hospitalization, comorbidity, long-term antibiotic treatment, and HAUTI caused by more varied and more resistant organisms.

In this study, *E. coli* was the commonest uropathogen, accounting for (53.9%) of CA-UTI and (37.0%) of HAUTI cases. This report is comparable with India [7], Bangladesh [18], and Ethiopia-Pawe and Hawassa [4,38]. Its prevalence may be due to virulence factors like P-fimbria and S-fimbria adhesins, which aid in urinary tract colonization and invasion [39]. However, *E. coli* was more prevalent in CA-UTI than HAUTI, consistent with studies from Bangladesh [5], and Pakistan [16]. This may be because UTI-causing bacteria primarily originate from fecal flora rather than hospital environments.

In the present study, *K. pneumoniae* was the second predominant bacteria isolated at a rate of (18.0% vs. 18.2%) in CA-UTI and HAUTI, respectively. This finding was a similar pattern with the study in India [7], and Palestine [16] in CA-UTI and HAUTI, respectively. But, this study prevalence was not correlated with other reports in Portugal CA-UTI (8.9%) [40], Saudi Arabia CA-UTI (8.1%) [41] and Ethiopia-Gondar CA-UTI and HAUTI (12.5% vs 25.0%) [42]. In our study, the prevalence of *Klebsiella* spp. in HAUTI (18.2%) was higher than in CA-UTI (18.0%). This report is similar with the study in Bangladesh [18] and Ethiopia-Pawe [4]. This can be justified by its ability for adaptation to the hospital environment, and it can survive longer than other bacteria, enabling cross-infection within hospitals [43].

In this study, *S. saprophyticus* and *S. aureus* were the third most common isolates, with prevalence rates of 14.6% in CA-UTI and 10.3% in HAUTI. However, these findings differ from reports in Turkey, where *P. aeruginosa* was common in HAUTI [44], and Sudan, where *E. faecalis* (13%) and *S. aureus* (10%) were the second and third most common CA-UTI isolates [12]. This variation may be due to the adaptability of Gram-positive bacteria to environmental factors like temperature, humidity, and resistance patterns. Additionally, increased catheter use in hospital settings could contribute to HAUTI cases [45]. *Proteus mirabilis* and *Citrobacter* spp.*,* were also identified in causing both CA-UTI and HAUTI. However, *K. rhinoscleromatis, Pseudomonas* spp., and *P. vulgaris* were isolated from hospitalized patients only. Similar results were found in Turkey [44] and Ethiopia-Addis Ababa [27]. *Pseudomonas* spp. is enabled to survive in hospital environment like sinks, water tanks, thrive well in soaps and disinfectants used for urethral catheterization [21].

Regarding antimicrobial susceptibility pattern, the Enterobacteriaceae in HAUTI were susceptible to piperacillin-tazobactam (100%), meropenem (84.7%), ciprofloxacin (71%), tetracycline (12.9%), and nalidixic acid (11.3%). Similar report is shown in Ethiopia-Dessie ciprofloxacin (69%), and tetracycline (17.2%) [21]. *Pseudomonas* spp. was susceptible to piperacillin-tazobactam (100%), meropenem (50%), and norfloxacin (25%). Gram-positive bacterial isolates were susceptible to gentamicin (82.8), trimethoprim-sulfamethoxazole (69%), vancomycin (58.6%), and penicillin (31%). The result of this study was lower than a study conducted in Ethiopia-Gondar gentamicin (92.9%) [23]. This difference may be attributed to variations in the study period, sample size, socio-demographic factors, and adherence to hand hygiene and catheter care practices.

Among CA-UTI cases, *E. coli, K. pneumoniae, P. mirabilis*, and *Citrobacter* spp. showed high susceptibility to piperacillin-tazobactam (100%), meropenem (97.1%), tetracycline (27.1%), and nalidixic acid (18.6%). It is similar to studies that were conducted in Iran meropenem (92.2%) [46] and Sudan piperacillin-tazobactam (100%) [12]. Gram-positive isolates were highly susceptible to gentamicin (100%), while vancomycin, and trimethoprim-sulfamethoxazole showed (68.4%) susceptibility, and penicillin (42.1%). These rates were higher than those reported in Iran gentamicin (64.4%) [46] and Ethiopia-Shashemene trimethoprim-sulfamethoxazole (46.6%) [25]. This could be due to lower antibiotic exposure to newer antibiotics, slower resistance spread, and reduced agricultural antibiotic use.

Comparatively, the antimicrobial susceptibility of majority of the isolates in HAUTI were resistant to tetracycline and nalidixic acid. But, in CA-UTI isolates were lower level of resistance observed with tetracycline and nalidixic acid. Similar pattern was shown in Ethiopia-Dessie [21]. This might be due to the empirical use of broad-spectrum antibiotics before definitive diagnosis with culture and susceptibility test has been made and transfer of antibiotic resistance gene from one bacterium to another bacterial species resulted in multiple antibiotic resistance.

## Conclusion

In this study, the overall prevalence rate of UTI was high, and the hospitalized geriatrics had a higher risk of developing bacteria UTI than the community geriatrics. The most important factors influencing UTIs are age and gender. *Escherichia coli* is the predominant cause of community and hospital-acquired UTI, along with its increasing resistance pattern to different antibiotics, and is going to be an alarming health problem. This study discovered that bacteria’s alarming level of resistance (nalidixic acid) is involved in UTI. In comparison to other antibiotics tested, the majority of the isolates were more sensitive to piperacillin-tazobactam and meropenem.

The healthcare policy should discourage unsafe catheterization, prolonged hospital stays (≥ 5 days), and prevent further development and distribution of more resistant uropathogens. Urinary tract infections in geriatric patients should be treated appropriately, avoiding the misuse of broad-spectrum antimicrobials as empirical treatment, due to the high prevalence of multiple antibiotic resistance in these patients. A continuous audit of the prevalence and antimicrobial susceptibility patterns of uropathogens in CA-UTI and HAUTI of geriatrics as a cause of morbidity should be performed, and the results should be reviewed on a regular basis. In general, it is imperative to create and enhance an awareness among the community members to prevent risk factors associated with the UTIs. Piperacillin-tazobactam could be used as a drug of choice for empirical treatment of UTI in this study area. Further intervention research targeting significantly associated risk factors is recommended to reduce the burden of UTI among geriatrics in the study area.

## Supporting information

S1 File Dataset. (XLSX)
